# The possibility of coexistence and co-development in language competition: ecology–society computational model and simulation

**DOI:** 10.1186/s40064-016-2482-0

**Published:** 2016-06-23

**Authors:** Jian Yun, Song-Chao Shang, Xiao-Dan Wei, Shuang Liu, Zhi-Jie Li

**Affiliations:** School of Computer Science and Engineering, Dalian Nationalities University, Dalian, 116600 Liaoning China; School of Computer Science and Engineering, University of Electronic Science and Technology of China, Chengdu, 610054 Sichuan China

**Keywords:** Language competition, Coexistence, Co-development, Computational model, Lotka–Volterra, Reaction–diffusion, Equilibria, Stability, Ecology–society

## Abstract

Language is characterized by both ecological properties and social properties, and competition is the basic form of language evolution. The rise and decline of one language is a result of competition between languages. Moreover, this rise and decline directly influences the diversity of human culture. Mathematics and computer modeling for language competition has been a popular topic in the fields of linguistics, mathematics, computer science, ecology, and other disciplines. Currently, there are several problems in the research on language competition modeling. First, comprehensive mathematical analysis is absent in most studies of language competition models. Next, most language competition models are based on the assumption that one language in the model is stronger than the other. These studies tend to ignore cases where there is a balance of power in the competition. The competition between two well-matched languages is more practical, because it can facilitate the co-development of two languages. A third issue with current studies is that many studies have an evolution result where the weaker language inevitably goes extinct. From the integrated point of view of ecology and sociology, this paper improves the Lotka–Volterra model and basic reaction–diffusion model to propose an “ecology–society” computational model for describing language competition. Furthermore, a strict and comprehensive mathematical analysis was made for the stability of the equilibria. Two languages in competition may be either well-matched or greatly different in strength, which was reflected in the experimental design. The results revealed that language coexistence, and even co-development, are likely to occur during language competition.

## Background

Research on language evolution is crucial for understanding the diversity of human culture. Over recent years, language evolution has become a popular topic in modern science. Evolution of language can largely be attributed to social behavior. Thus, the factor of social behavior is one of the motivations of language evolution. The original meaning of the word evolution is the difference in living beings between generations, even including competition amongst species. American linguist Mufwene (2001) considers language a biological species and analyzes language evolution from a brand-new ecological point of view, thus formulating language evolution ecology. Mufwene (2001) describes language as being like a parasite on a host, which is the talking person. Here, competition and selection are the basic evolution modes of language species evolution, and the rise and decline of any given language is the result of language competition. Language evolution is characterized by language competition, while language competition is characterized by both ecological properties and social properties.

This paper proposes an “ecology–society” computational model that describes the coexistence and co-development within language competition. The model is then used to prove the possibility of the coexistence of two competitive languages.

The remainder of this paper is organized as follows. The “Related work” section presents related research. The “Stability of equilibria in associated computational models” section contains the mathematical analysis of equilibria and their stability in an existing computation model. The “Ecology–society computational model” section describes the proposed “ecology–society” computational model for language competition, along with a relatively strict mathematical analysis and theoretical arguments. The “Experiments and analysis” section shows the experiments that were designed to prove the validity of the model. Finally, the “Conclusion” section presents the conclusion.

## Related work

Research on language evolution can be divided into two categories: language origin and change, and language competition. The former is usually carried out in an interdisciplinary manner, with relatively obvious cross characterizations of linguistics, evolutionary biology, nerve and brain function science, informetrics, and computer science. Research regarding language competition, on the other hand, is typically considered to be more practical, as various models produced in language competition research can provide a quantitative evaluation and estimation for the tendency of the two languages in competition.

The various components of language origin and change have made significant contributions to the field. In terms of linguistics, the language variation theories presented by Labov (1994, 2001) are considered standard in language evolution. Studies in the field of evolutionary biology have also made useful contributions to language evolution research. The findings of Allentoft et al. (2015), Novembre (2015) are consistent with the hypothesized spread of Indo-European languages during the Early Bronze Age, and the genome-wide DNA analysis of Haak et al. (2015), Novembre (2015) showed that the massive migration from the steppe was a source for Indo-European languages in Europe. Nerve and brain function science studies have demonstrated that human brain specializations that support language can be identified by comparing human brains with non-human primate brains; the volume of human arcuate fasciculus is much larger than other primates, and only human arcuate fasciculus completely connect the Broca area in the left brain prefrontal cortex with the Wernicke area behind the temporal lobeand (Rilling et al. 2008; Rilling 2014). These two brain regions are both language function areas. Informetrics have also been introduced in this research branch. Petersen et al. (2012) showed a decreasing marginal need for new words in language expansion by analyzing the occurrence frequencies of over 15 million words in seven different languages. Perc (2012) studied the evolution of the most common English words and phrases over the centuries based on statistical analysis and contemporary network science. Perc (2014) also studied the Matthew effect in social phenomena and emphasized the importance of cumulative advantage processes in language change. Computer science has also presented relevant results in recent years by considering the formation of language structure as an entry point. Here, language structure refers to phonetics, syntax, and vocabulary. In human language systems, phonetics, syntax, and vocabulary are computable. Investigators have created a wide variety of research in the field of computer science regarding language evolution: Ke et al. (2003) proposed a human vowel system and tone system optimization model based on genetic algorithms; Mukherjee et al. (2007) proposed a human consonant system co-occurrence model based on community finding algorithms in complex networks (Radicchi et al. 2004); Redford et al. (2001) modeled the emergence of human syllable systems based on symbiotic evolutionary algorithms; Shaobai et al. (2015) investigated the mechanism for phonating stressed English syllables based on an improved neural network model; De and Zuidema (2010) investigated the evolution of combinatorial phonology with a multi-agents model; Gong (2011) presented a syntactic model that discusses the process of universal, non-language-specific mechanisms that help individuals acquire vocabulary and syntax; and Kirby et al. (2014) reviewed various methods, such as computational agent-based simulations and mathematical modeling, for understanding how behavior is shaped by the iterated learning process, and then showed how an iterated learning framework has been used to explain the origins of structure in language.

In recent years, language competition has become an increasingly popular topic in language evolution research. This branch primarily focuses on computer science, mathematics, and ecology. The language competition dynamics model proposed by Abrams and Strogatz (2003) is considered to be the standard in this branch. The AS (Abrams-Strogatz) model suggests that the coexistence of two languages competition is not stable and that the weaker one will eventually become extinct. The parameter *s* in this model represents the social state of the language. Other researchers (Stauffer et al. 2007; Caridi et al. 2013) have considered the AS model and further discussed the phenomenon of language competition by using an agent-based method. Patriarca and Leppänen (2004) discussed the coexistence of two languages in two disjoint zones using a reaction–diffusion equation. However, this research is not suitable for the coexistence of two languages in competition in one zone. Pinasco and Romanelli (2006) combined the famous species competition model, also known as the Lotka–Volterra model, with the infectious disease model to propose a new ecological model that is capable of explaining the coexistence of two competitive languages in one zone. Based on this work, Kandler and Steele (2008) took common carrying capacity and spatial heterogeneity into account and improved the ecological model. Zhang and Gong (2013) discussed the rules in qualitative and quantitative analysis of various parameters in the language competition model. Overall, both ecological modeling and sociological modeling are useful methods for studying language evolution.

It is highly important to develop a computational model that integrates both ecological and societal characteristics, as language can be classified by both ecological species and social status. In fact, mathematical modeling of competition in “ecology–societal” models such as sustainable development, population growth and cultural evolution has been widely discussed (Perc and Szolnoki 2010; Ghirlanda et al. 2010). In addition, many existing language competition modeling studies are driven by endangered language extinction crises; the associated models tend to assume that one language is weaker than the other in advance. However, competition between two well-matched languages is more fruitful as a subject of research. If the two languages in competition are evenly matched, the competition is more valuable, as it is less predictable. The core value here is that the two languages can develop jointly through competition. Research on language competition will be more valuable if it is more applicable to a real world setting.

## Stability of equilibria in associated computational models

Consider the two following differential equations defined as Theorem (Xue 2011):

$$\begin{aligned} \frac{{{\text{d}}x_{1} }}{{{\text{d}}t}} = f_{ 1} (x_{1} ,x_{2} ), \hfill \\ \frac{{{\text{d}}x_{2} }}{{{\text{d}}t}} = f_{ 2} (x_{1} ,x_{2} ) \hfill \\ \end{aligned}$$

The corresponding algebraic equations are:

$$\begin{aligned} f_{ 1} (x_{1} ,x_{2} ) = 0, \hfill \\ f_{ 2} (x_{1} ,x_{2} ) = 0. \hfill \\ \end{aligned}$$

The real roots $x_{1} = x_{1}^{*} ,x_{2} = x_{2}^{*}$ of the algebraic equations are the equilibria of the mentioned differential equations, and they are denoted by ($x_{1}^{*}$, $x_{2}^{*}$).

Let *A*, *p*, and *q* be defined by the following equations:

$$A = \left[ {\begin{array}{*{20}c} {\frac{{\partial f_{1} (x_{1}^{*} ,x_{2}^{*} )}}{{\partial x_{1} }}} & {\frac{{\partial f_{1} (x_{1}^{*} ,x_{2}^{*} )}}{{\partial x_{2} }}} \\ {\frac{{\partial f_{2} (x_{1}^{*} ,x_{2}^{*} )}}{{\partial x_{1} }}} & {\frac{{\partial f_{2} (x_{1}^{*} ,x_{2}^{*} )}}{{\partial x_{2} }}} \\ \end{array} } \right],$$

$$p = - \left( {\frac{{\partial f_{1} (x_{1}^{*} ,x_{2}^{*} )}}{{\partial x_{1} }} + \frac{{\partial f_{2} (x_{1}^{*} ,x_{2}^{*} )}}{{\partial x_{2} }}} \right),$$

$$q = \frac{{\partial f_{1} (x_{1}^{*} ,x_{2}^{*} )}}{{\partial x_{1} }} \cdot \frac{{\partial f_{2} (x_{1}^{*} ,x_{2}^{*} )}}{{\partial x_{2} }} - \frac{{\partial f_{2} (x_{1}^{*} ,x_{2}^{*} )}}{{\partial x_{1} }} \cdot \frac{{\partial f_{1} (x_{1}^{*} ,x_{2}^{*} )}}{{\partial x_{2} }}$$

If *p* > 0 and *q* > 0, then ($x_{1}^{*}$, $x_{2}^{*}$) is stable. If *p* < 0 or *q* < 0, then ($x_{1}^{*}$, $x_{2}^{*}$) is unstable.

### Lotka–Volterra/infectious disease hybrid model and basic reaction–diffusion model

The hybrid model of Lotka–Volterra and infectious disease, proposed by Pinasco and Romanelli (2006), is as follows:

1 $$\begin{aligned} \frac{{\partial u_{1} }}{\partial t} & = cu_{1} u_{2} + a_{1} u_{1} \left( {1 - \frac{{u_{1} }}{{K_{1} }}} \right) \\ \frac{{\partial u_{2} }}{\partial t} & = - cu_{1} u_{2} + a_{2} u_{2} \left( {1 - \frac{{u_{2} }}{{K_{2} }}} \right) \\ \end{aligned}$$

The basic reaction–diffusion language competition model, proposed by Kandler and Steele (2008), is as follows:

2 $$\begin{aligned} \frac{{\partial u_{1} }}{\partial t} & = d_{1} \Delta u_{1} + u_{1} \left[ {a_{1} - \frac{{a_{1} }}{{K_{1} }}u_{1} + cu_{2} } \right] \\ \frac{{\partial u_{2} }}{\partial t} & = d_{2} \Delta u_{2} + u_{2} \left[ {a_{2} - \frac{{a_{2} }}{{K_{2} }}u_{2} - cu_{1} } \right] \\ \end{aligned}$$

where $u_{1}$ and $u_{2}$ represent the frequencies of the populations using language 1 and language 2, respectively; $a_{1}$ and $a_{2}$ represent the growth rate of the number of speakers who speak language 1 and language 2, respectively; $K_{1}$ and $K_{2}$ represent the carrying capacities of the populations using language 1 and language 2, respectively; $c$ is the attractiveness of language 1 to language 2, which can also be defined as the conversion rate of language 2 to language 1; $d_{1} \Delta u_{1}$ and $d_{2} \Delta u_{2}$ represent the diffusion components of the populations using language 1 and language 2, respectively; and $\Delta$ is the Laplace operator.

The equilibria and stabilities of models (1) and (2) are equal according to the following detailed analysis.

The four equilibrium points can be determined according to Theorem (Xue 2011):

$$(u_{1}^{*} ,u_{2}^{*} ) = (0,0),$$

$$(u_{1}^{*} ,u_{2}^{*} ) = (K_{1} ,0),$$

$$(u_{1}^{*} ,u_{2}^{*} ) = (0,K_{2} ),$$

$$(u_{1}^{*} ,u_{2}^{*} ) = \left( {\frac{{a_{2} K_{1} (a_{1} + cK_{2} )}}{{a_{1} a_{2} + c^{2} K_{1} K_{2} }},\frac{{a_{1} K_{2} (a_{2} - cK_{1} )}}{{a_{1} a_{2} + c^{2} K_{1} K_{2} }}} \right).$$

For the equilibrium to be positive, $K_{1} < \frac{{a_{2} }}{c}$ must be satisfied. The following can be defined according to Theorem (Xue 2011):

$$A = \left[ {\begin{array}{*{20}c} {a_{1} - \frac{{2a_{1} u_{1} }}{{K_{1} }} + cu_{2} } & {cu_{1} } \\ { - cu_{2} } & {a_{2} - cu_{1} - \frac{{2a_{2} u_{2} }}{{K_{2} }}} \\ \end{array} } \right]$$

For the equilibrium $(u_{1}^{*} ,u_{2}^{*} ) = (0,0)$, matrix $$A = \left[ {\begin{array}{*{20}c} {a_{1} } & 0 \\ 0 & {a_{2} } \\ \end{array} } \right]$$. Equilibrium $(u_{1}^{*} ,u_{2}^{*} ) = (0,0)$ is not stable, because $p = - (a_{1} + a_{2} ) < 0$. For the equilibrium $(u_{1}^{*} ,u_{2}^{*} ) = (0,K_{2} )$, matrix $$A = \left[ {\begin{array}{*{20}c} {a_{1} + cK_{2} } & 0 \\ { - cK_{2} } & { - a_{2} } \\ \end{array} } \right]$$. The equilibrium $(u_{1}^{*} ,u_{2}^{*} ) = (0,K_{2} )$ is not stable, because $q = - a_{2} (a_{1} + cK_{2} ) < 0$. For the equilibrium $(u_{1}^{*} ,u_{2}^{*} ) = (K_{1} ,0)$, matrix $$A = \left[ {\begin{array}{*{20}c} { - a_{1} } & {cK_{1} } \\ 0 & {a_{2} - cK_{1} } \\ \end{array} } \right]$$. When the premise condition $K_{1} < \frac{{a_{2} }}{c}$ is satisfied, $q < 0$ because $q = - a_{1} (a_{2} - cK_{1} )$. Thus, the equilibrium $(u_{1}^{*} ,u_{2}^{*} ) = (K_{1} ,0)$ is not stable. For the equilibrium $(u_{1}^{*} ,u_{2}^{*} ) = \left( {\frac{{a_{2} K_{1} (a_{1} + cK_{2} )}}{{a_{1} a_{2} + c^{2} K_{1} K_{2} }},\frac{{a_{1} K_{2} (a_{2} - cK_{1} )}}{{a_{1} a_{2} + c^{2} K_{1} K_{2} }}} \right)$, matrix $$A = \left[ {\begin{array}{*{20}c} { - \frac{{a_{1} a_{2} (a_{1} + cK_{2} )}}{{a_{1} a_{2} + c^{2} K_{1} K_{2} }}} & {\frac{{ca_{2} K_{1} (a_{1} + cK_{2} )}}{{a_{1} a_{2} + c^{2} K_{1} K_{2} }}} \\ {\frac{{a_{1} cK_{2} (cK_{1} - a_{2} )}}{{a_{1} a_{2} + c^{2} K_{1} K_{2} }}} & {\frac{{a_{1} a_{2} (cK_{1} - a_{2} )}}{{a_{1} a_{2} + c^{2} K_{1} K_{2} }}} \\ \end{array} } \right]$$. When $K_{1} < \frac{{a_{2} }}{c}$, $p > 0,\;q > 0$ because $p = \frac{{a_{1} a_{2} (a_{1} + a_{2} )}}{{a_{1} a_{2} + c^{2} K_{1} K_{2} }},$ and $q = - \frac{{a_{1} a_{2} (a_{1} + cK_{2} )(cK_{1} - a_{2} )}}{{a_{1} a_{2} + c^{2} K_{1} K_{2} }}$. Here, the equilibrium $(u_{1}^{*} ,u_{2}^{*} ) = \left( {\frac{{a_{2} K_{1} (a_{1} + cK_{2} )}}{{a_{1} a_{2} + c^{2} K_{1} K_{2} }},\frac{{a_{1} K_{2} (a_{2} - cK_{1} )}}{{a_{1} a_{2} + c^{2} K_{1} K_{2} }}} \right)$ is stable.

Based on the above analysis of the four equilibria, it can be seen that when $K_{1} < \frac{{a_{2} }}{c}$, the only stable equilibrium is $(u_{1}^{*} ,u_{2}^{*} ) = \left( {\frac{{a_{2} K_{1} (a_{1} + cK_{2} )}}{{a_{1} a_{2} + c^{2} K_{1} K_{2} }},\frac{{a_{1} K_{2} (a_{2} - cK_{1} )}}{{a_{1} a_{2} + c^{2} K_{1} K_{2} }}} \right)$, which allows for the possibility of language coexistence.

### Reaction–diffusion model with common carrying capacity

Kandler and Steele (2008) proposed the reaction–diffusion language competition model with common carrying capacity:

3 $$\begin{aligned} \frac{{\partial u_{1} }}{\partial t} = d_{1} \Delta u_{1} + a_{1} u_{1} \left( {1 - \frac{{u_{1} }}{{K - u_{2} }}} \right) + cu_{1} u_{2} \hfill \\ \frac{{\partial u_{2} }}{\partial t} = d_{2} \Delta u_{2} + a_{2} u_{2} \left( {1 - \frac{{u_{2} }}{{K - u{}_{1}}}} \right) - cu_{1} u_{2} \hfill \\ \end{aligned}$$

where *K* represents the maximum carrying capacity of the sum of the populations using language 1 and language 2.

The following five equilibria can be defined according to Theorem (Xue 2011):

$$(u_{1}^{*} ,u_{2}^{*} ) = (0,0),$$

$$(u_{1}^{*} ,u_{2}^{*} ) = (K,0),$$

$$(u_{1}^{*} ,u_{2}^{*} ) = (0,K),$$

$$(u_{1}^{*} ,u_{2}^{*} ) = \left( {\frac{{cK + 2a_{2} + (c^{2} K^{2} - 4a_{1} a_{2} )^{1/2} }}{2c},\frac{{cK - 2a_{1} + (c^{2} K^{2} - 4a_{1} a_{2} )^{1/2} }}{2c}} \right),$$

$$(u_{1}^{*} ,u_{2}^{*} ) = \left( {\frac{{cK + 2a_{2} - (c^{2} K^{2} - 4a_{1} a_{2} )^{1/2} }}{2c},\frac{{cK - 2a_{1} - (c^{2} K^{2} - 4a_{1} a_{2} )^{1/2} }}{2c}} \right).$$

To keep the fourth equilibrium positive, $c^{2} K^{2} > 4a_{1} a_{2} ,cK - 2a_{1} + (c^{2} K^{2} - 4a_{1} a_{2} )^{1/2} > 0$ must be satisfied, so $K > \frac{{2(a_{1} a_{2} )^{1/2} }}{c}\;\;\;{\text{and}}\;\;\;a_{2} > a_{1}$.

To keep the fifth equilibrium positive, $c^{2} K^{2} > 4a_{1} a_{2} ,cK - 2a_{1} - (c^{2} K^{2} - 4a_{1} a_{2} )^{1/2} > 0$ must be satisfied, so $\frac{{a_{1} + a_{2} }}{c} > K > \frac{{2(a_{1} a_{2} )^{1/2} }}{c}$.

The common carrying requirement of the fourth and fifth equilibria must be verified, that is, $u_{1} + u_{2} \le K$.

For the fourth equilibrium,

$$\begin{aligned} u_{1}^{*} + u_{2}^{*} & = \frac{{cK + 2a_{2} + (c^{2} K^{2} - 4a_{1} a_{2} )^{1/2} + cK - 2a_{1} + (c^{2} K^{2} - 4a_{1} a_{2} )^{1/2} }}{2c} \\ & = K + \frac{{a_{2} - a_{1} + (c^{2} K^{2} - 4a_{1} a_{2} )^{1/2} }}{c}. \\ \end{aligned}$$

because $$a_{2} > a_{1}$$, $$u_{1}^{*} + u_{2}^{*} > K$$.

For the fifth equilibrium, $u_{1}^{*} + u_{2}^{*} = K + \frac{{a_{2} - a_{1} - (c^{2} K^{2} - 4a_{1} a_{2} )^{1/2} }}{c}$.

$${\text{If}}\; a_{2} < a_{1} ,\;u_{1}^{*} + u_{2}^{*} < K;$$

$${\text{If}}\;a_{2} > a_{1} ,\;\frac{{a_{1} + a_{2} }}{c} > K > \frac{{2(a_{1} a_{2} )^{1/2} }}{c};$$

$${\text{If}}\;K = \frac{{2(a_{1} a_{2} )^{1/2} }}{c},\;u_{1}^{*} + u_{2}^{*} = K + \frac{{a_{2} - a_{1} }}{c} > K;$$

$${\text{If}}\;K = \frac{{a_{1} + a_{2} }}{c},\;u_{1}^{*} + u_{2}^{*} = K.$$

Assuming $\phi (x) = x + \frac{{a_{2} - a_{1} - (c^{2} x^{2} - 4a_{1} a_{2} )^{1/2} }}{c},$ then $\phi^{'} (x) = 1 - \frac{cx}{{(c^{2} x^{2} - 4a_{1} a_{2} )^{1/2} }} < 0,$ so $\phi (x)$ is monotonically decreasing. Based on the properties of the monotonic decreasing function, if $\frac{{a_{1} + a_{2} }}{c} > k > \frac{{2(a_{1} a_{2} )^{1/2} }}{c}$, then $u_{1}^{*} + u_{2}^{*} \ge K$.

According to Theorem (Xue 2011),

$$A = \left[ {\begin{array}{*{20}c} {a_{1} - \frac{{2a_{1} u_{1} }}{{K - u_{2} }} + cu_{2} } & {cu_{1} - \frac{{a_{1} u_{1}^{2} }}{{(K - u_{2} )^{2} }}} \\ { - cu_{2} - \frac{{a_{2} u_{2}^{2} }}{{(K - u_{1} )^{2} }}} & {a_{2} - cu_{1} - \frac{{2a_{2} u_{2} }}{{K - u_{1} }}} \\ \end{array} } \right]$$

For the equilibrium $(u_{1}^{*} ,u_{2}^{*} ) = (0,0)$ ,matrix $$A = \left[ {\begin{array}{*{20}c} {a_{1} } & 0 \\ 0 & {a_{2} } \\ \end{array} } \right]$$. Equilibrium $(u_{1}^{*} ,u_{2}^{*} ) = (0,0)$ is not stable, because $p = - (a_{1} + a_{2} ) < 0$.

For the equilibrium $(u_{1}^{*} ,u_{2}^{*} ) = (0,K)$, matrix $$A = \left[ {\begin{array}{*{20}c} {a_{1} + cK} & 0 \\ { - cK - a_{2} } & { - a_{2} } \\ \end{array} } \right]$$. Equilibrium $(u_{1}^{*} ,u_{2}^{*} ) = (0,K)$ is not stable, because $q = - a_{2} (a_{1} + cK) < 0$.

For the equilibrium $(u_{1}^{*} ,u_{2}^{*} ) = (K,0)$ ,matrix $$A = \left[ {\begin{array}{*{20}c} { - a_{1} } & {cK - a_{1} } \\ 0 & {a_{2} - cK} \\ \end{array} } \right]$$, and $p = - ( - a_{1} + a_{2} - cK),q = - a_{1} (a_{2} - cK)$ when $K < \frac{{a_{2} }}{c}$. Here, the equilibrium is not stable, because $q < 0$. When $K > \frac{{a_{2} }}{c}$, the equilibrium is stable, because $p > 0,q > 0$.

For the equilibrium $(u_{1}^{*} ,u_{2}^{*} ) = \left( {\frac{{cK + 2a_{2} + (c^{2} K^{2} - 4a_{1} a_{2} )^{1/2} }}{2c},\frac{{cK - 2a_{1} + (c^{2} K^{2} - 4a_{1} a_{2} )^{1/2} }}{2c}} \right),$

because $p = - \left( {a_{1} - \frac{{2a_{1} u_{1} }}{{K - u_{2} }} + cu_{2} + a_{2} - cu_{1} - \frac{{2a_{2} u_{2} }}{{K - u_{1} }}} \right) = 0$, $(u_{1}^{*} ,u_{2}^{*} ) = \left( {\frac{{cK + 2a_{2} + (c^{2} K^{2} - 4a_{1} a_{2} )^{1/2} }}{2c},\frac{{cK - 2a_{1} + (c^{2} K^{2} - 4a_{1} a_{2} )^{1/2} }}{2c}} \right)$ is not stable.

For the equilibrium $(u_{1}^{*} ,u_{2}^{*} ) = \left( {\frac{{cK + 2a_{2} - (c^{2} K^{2} - 4a_{1} a_{2} )^{1/2} }}{2c},\frac{{cK - 2a_{1} - (c^{2} K^{2} - 4a_{1} a_{2} )^{1/2} }}{2c}} \right)$, because $p = - \left( {a_{1} - \frac{{2a_{1} u_{1} }}{{K - u_{2} }} + cu_{2} + a_{2} - cu_{1} - \frac{{2a_{2} u_{2} }}{{K - u_{1} }}} \right) = 0$, the equilibrium $(u_{1}^{*} ,u_{2}^{*} ) = \left( {\frac{{cK + 2a_{2} - (c^{2} K^{2} - 4a_{1} a_{2} )^{1/2} }}{2c},\frac{{cK - 2a_{1} - (c^{2} K^{2} - 4a_{1} a_{2} )^{1/2} }}{2c}} \right)$ is not stable.

The above analysis demonstrates that the introduction of the common carrying capacity alone is not sufficient to make the languages coexist. The fourth and fifth equilibria are neither stable nor able to meet the requirements $cu_{1} u_{2}$ in most cases. In other words, the common carrying capacity does not set the upper limit of the sum of the two components for one equilibrium. This shows that the model is weaker than both the Lotka–Volterra model and the basic reaction–diffusion model for describing language coexistence. The model describes the language coexistence mainly by regulating the diffusion coefficient in the reaction–diffusion equation. The basic reaction–diffusion model also utilizes the diffusion coefficient.

In addition, each of the three models mentioned above implies that language 1 is stronger than language 2. The reason is that there is $cu_{1} u_{2}$ in language 1, $cu_{1} u_{2}$ in language 2, and *c* > 0 in the model. None of the three models successfully describe more valuable, more common, and more real competition, which is defined by co-development in competition.

## Ecology–society computational model

### Proposal and description of the model

This paper proposes a model that offers an improvement upon the Lotka–Volterra model and the basic reaction–diffusion model based on the analysis of the various models in the previous section. These two models were chosen for improvement due to their ability to describe language co-existence. The improved model is therefore expected to describe both language coexistence and language co-development. The new model reflects both the ecological elements that the language as a species should have and the social status elements of the language. Equation 4 shows the improved model:

4 $$\begin{aligned} \frac{{\partial u_{1} }}{\partial t} = d_{1} \Delta u_{1} + a_{1} u_{1} \left( {1 - \frac{{u_{1} }}{{K_{1} }} - \frac{{\alpha u_{2} }}{{K_{1} }}} \right) + c_{1} u_{1} u_{2} \hfill \\ \frac{{\partial u_{2} }}{\partial t} = d_{2} \Delta u_{2} + a_{2} u_{2} \left( {1 - \frac{{u_{2} }}{{K_{2} }} - \frac{{\beta u_{1} }}{{K_{2} }}} \right) + c_{2} u_{1} u_{2} \hfill \\ \end{aligned}$$

where $d_{1} \Delta u_{1}$ and $d_{2} \Delta u_{2}$ represent the diffusion terms of the populations using languages 1 and language 2, respectively; $a_{1} u_{1} \left( {1 - \frac{{u_{1} }}{{K_{1} }} - \frac{{\alpha u_{2} }}{{K_{1} }}} \right)$ and $a_{2} u_{2} \left( {1 - \frac{{u_{2} }}{{K_{2} }} - \frac{{\beta u_{1} }}{{K_{2} }}} \right)$ reflect the characteristics of the Lotka–Volterra model and represent the change of language frequency influenced by the population’s inherent growth rate, the competition within the population, and the competition among populations using language 1 and language 2, respectively; $c_{1}$ is the attractiveness of language 1 to language 2, which can also be described as the conversion rate representing the percentage of people turning to speak language 1 in the population using language 2; and $c_{2}$ is the attractiveness of language 2 to language 1. In this model, the role of parameters $c_{1}$ and $c_{2}$ is similar to parameter *s* in the AS model (Abrams and Strogatz 2003). They are used to represent the social combination factors affecting language competition, including language position, media influence, geographical distribution of the language-speaking population, and domestic policy on the language. In contrast to existing models that consider one language to be stronger than the other, the current model focuses on the co-development of two well-matched languages in competition. In the model, there is $c_{1} u_{1} u_{2}$ in language 1, $c_{2} u_{1} u_{2}$ in language 2, and $c_{1}$ > 0 and $c_{2}$ > 0.

### Analysis of equilibria and their stabilities

The following four equilibria can be determined according to Theorem (Xue 2011):

$$(u_{1}^{*} ,u_{2}^{*} ) = (0,0),$$

$$(u_{1}^{*} ,u_{2}^{*} ) = (K_{1} ,0),$$

$$(u_{1}^{*} ,u_{2}^{*} ) = (0,K_{2} ),$$

$$(u_{1}^{ * } ,u_{2}^{ * } ) = \left( {\frac{{a_{2} (K_{2} \alpha a_{1} - K_{2} c_{1} K_{1} - a_{1} K_{1} )}}{{ - a_{1} a_{2} + \alpha \beta a_{1} a_{2} - \beta a_{2} c_{1} K_{1} - c_{2} K_{2} \alpha a_{1} + c_{2} K_{2} c_{1} K_{1} }},\frac{{a_{1} (K_{1} \beta a_{2} - K_{2} c_{2} K_{1} - a_{2} K_{2} )}}{{ - a_{1} a_{2} + \alpha \beta a_{1} a_{2} - \beta a_{2} c_{1} K_{1} - c_{2} K_{2} \alpha a_{1} + c_{2} K_{2} c_{1} K_{1} }}} \right)$$

#### Positive analysis of equilibria

This section presents mathematical analysis of the requirements to keep the fourth equilibrium positive.

1. Assume $- a_{1} a_{2} + \alpha \beta a_{1} a_{2} - \beta a_{2} c_{1} K_{1} - c_{2} K_{2} \alpha a_{1} + c_{2} K_{2} c_{1} K_{1} > 0$, which is equivalent to $c_{1} K_{1} (c_{2} K_{2} - \beta a_{2} ) > a_{1} (c_{2} K_{2} \alpha + a_{2} - \alpha \beta a_{2} )$. Thus, $K_{1} > \frac{{a_{1} (c_{2} K_{2} \alpha + a_{2} - \alpha \beta a_{2} )}}{{c_{1} (c_{2} K_{2} - \beta a_{2} )}} > 0$ when $c_{2} K_{2} - \beta a_{2} > 0$, and $0 < K_{1} < \frac{{a_{1} (c_{2} K_{2} \alpha + a_{2} - \alpha \beta a_{2} )}}{{c_{1} (c_{2} K_{2} - \beta a_{2} )}}$ when $c_{2} K_{2} - \beta a_{2} < 0$.

In the first case, $K_{1} \beta a_{2} - K_{2} c_{2} K_{1} - a_{2} K_{2} > 0$. This is because the numerator must be greater than zero if the denominator is greater than zero. Therefore, $K_{1} < \frac{{a_{2} K_{2} }}{{\beta a_{2} - c_{2} K_{2} }} < 0$, which is obviously not plausible.

In the second case, $K_{2} \alpha a_{1} - K_{2} c_{1} K_{1} - a_{1} K_{1} > 0$. Here, $K_{1} < \frac{{K_{2} \alpha a_{1} }}{{K_{2} c_{1} + a_{1} }}$. Since $K_{1} \beta a_{2} - K_{2} c_{2} K_{1} - a_{2} K_{2} > 0$, $K_{1} > \frac{{a_{2} K_{2} }}{{\beta a_{2} - K_{2} c_{2} }}$.

Assuming that the denominator of the equilibrium is greater than zero, $\frac{{a_{2} K_{2} }}{{\beta a_{2} - K_{2} c_{2} }} < K_{1} < \hbox{min} \left( {\frac{{K_{2} \alpha a_{1} }}{{K_{2} c_{1} + a_{1} }},\frac{{a_{1} (c_{2} K_{2} \alpha + a_{2} - \alpha \beta a_{2} )}}{{c_{1} (c_{2} K_{2} - \beta a_{2} )}}} \right)$ must be satisfied.

2. Assume $- a_{1} a_{2} + \alpha \beta a_{1} a_{2} - \beta a_{2} c_{1} K_{1} - c_{2} K_{2} \alpha a_{1} + c_{2} K_{2} c_{1} K_{1} < 0$, which is equivalent to $c_{1} K_{1} (c_{2} K_{2} - \beta a_{2} ) < a_{1} (c_{2} K_{2} \alpha + a_{2} - \alpha \beta a_{2} )$. Therefore, $0 < K_{1} < \frac{{a_{1} (c_{2} K_{2} \alpha + a_{2} - \alpha \beta a_{2} )}}{{c_{1} (c_{2} K_{2} - \beta a_{2} )}}$ when $c_{2} K_{2} - \beta a_{2} > 0$, and $K_{1} > \frac{{a_{1} (c_{2} K_{2} \alpha + a_{2} - \alpha \beta a_{2} )}}{{c_{1} (c_{2} K_{2} - \beta a_{2} )}} > 0$ when $c_{2} K_{2} - \beta a_{2} < 0$.

In the first case, $K_{1} \beta a_{2} - K_{2} c_{2} K_{1} - a_{2} K_{2} < 0$. This is because the numerator must be less than zero if the denominator is less than zero. Then, $K_{1} > 0 > \frac{{a_{2} K_{2} }}{{\beta a_{2} - c_{2} K_{2} }}$. Since $K_{2} \alpha a_{1} - K_{2} c_{1} K_{1} - a_{1} K_{1} < 0$, then $K_{1} > \frac{{K_{2} \alpha a_{1} }}{{K_{2} c_{1} + a_{1} }}$.

In the second case, $K_{2} \alpha a_{1} - K_{2} c_{1} K_{1} - a_{1} K_{1} < 0$, $K_{1} > \frac{{K_{2} \alpha a_{1} }}{{K_{2} c_{1} + a_{1} }}$. Since $K_{1} \beta a_{2} - K_{2} c_{2} K_{1} - a_{2} K_{2} < 0$, $K_{1} < \frac{{a_{2} K_{2} }}{{\beta a_{2} - K_{2} c_{2} }}$.

Assuming that the denominator of the equilibrium is smaller than zero, $\hbox{max} \;\left( {\frac{{a_{1} (c_{2} K_{2} \alpha + a_{2} - \alpha \beta a_{2} )}}{{c_{1} (c_{2} K_{2} - \beta a_{2} )}},\frac{{K_{2} \alpha a_{1} }}{{K_{2} c_{1} + a_{1} }}} \right) < K_{1} < \frac{{a_{2} K_{2} }}{{\beta a_{2} - K_{2} c_{2} }}$ or $\frac{{K_{2} \alpha a_{1} }}{{K_{2} c_{1} + a_{1} }} < K_{1} < \frac{{a_{1} (c_{2} K_{2} \alpha + a_{2} - \alpha \beta a_{2} )}}{{c_{1} (c_{2} K_{2} - \beta a_{2} )}}$ must be satisfied.

#### Analysis of the stablities of the equilibria

According to Theorem (Xue 2011),

$$A = \left[ {\begin{array}{*{20}c} {a_{1} - \frac{{2a_{1} u_{1} }}{{K_{1} }} - \frac{{\alpha a_{1} u_{2} }}{{K_{1} }} + c_{1} u_{2} } & {c_{1} u_{1} - \frac{{\alpha a_{1} u_{1} }}{{K_{1} }}} \\ {c_{2} u_{2} - \frac{{\beta a_{2} u_{2} }}{{K_{2} }}} & {a_{2} - \frac{{2a_{2} u_{2} }}{{K_{2} }} - \frac{{\beta a_{2} u_{1} }}{{K_{2} }} + c_{2} u_{1} } \\ \end{array} } \right].$$

For the equilibrium $(u_{1}^{*} ,u_{2}^{*} ) = (0,0)$, matrix $$A = \left[ {\begin{array}{*{20}c} {a_{1} } & 0 \\ 0 & {a_{2} } \\ \end{array} } \right]$$. Since $p = - (a_{1} + a_{2} ) < 0,$ equilibrium $(u_{1}^{*} ,u_{2}^{*} ) = (0,0)$ is not stable.

For the equilibrium $(u_{1}^{*} ,u_{2}^{*} ) = (0,K_{2} )$,

$$\begin{aligned} p & = - \left( {a_{1} - \frac{{2a_{1} u_{1} }}{{K_{1} }} - \frac{{\alpha a_{1} u_{2} }}{{K_{1} }} + c_{1} u_{2} + a_{2} - \frac{{2a_{2} u_{2} }}{{K_{2} }} - \frac{{\beta a_{2} u_{1} }}{{K_{2} }} + c_{2} u_{1} } \right) = a_{2} - a_{1} + \frac{{\alpha a_{1} K_{2} }}{{K_{1} }} - c_{1} K_{2} \\ q & = \left( {a_{1} - \frac{{2a_{1} u_{1} }}{{K_{1} }} - \frac{{\alpha a_{1} u_{2} }}{{K_{1} }} + c_{1} u_{2} } \right)\left( {a_{2} - \frac{{2a_{2} u_{2} }}{{K_{2} }} - \frac{{\beta a_{2} u_{1} }}{{K_{2} }} + c_{2} u_{1} } \right) - \left( {c_{1} u_{1} - \frac{{\alpha a_{1} u_{1} }}{{K_{1} }}} \right)\left( {c_{2} u_{2} - \frac{{\beta a_{2} u_{2} }}{{K_{2} }}} \right) \\ & = - a_{2} \left( {a_{1} - \frac{{\alpha a_{1} k_{2} }}{{k_{1} }} + c_{1} k_{2} } \right) \\ \end{aligned}$$

According to Theorem (Xue 2011), since $p > 0$ and ${\text{q}} > 0$, $K_{1} < \frac{{\alpha a_{1} K_{2} }}{{a_{1} + c_{1} K_{2} }}$. Therefore, equilibrium $(u_{1}^{*} ,u_{2}^{*} ) = (0,K_{2} )$ is stable when $K_{1} < \frac{{\alpha a_{1} K_{2} }}{{a_{1} + c_{1} K_{2} }}$.

For the equilibrium $(u_{1}^{*} ,u_{2}^{*} ) = (K_{1} ,0)$,

$$\begin{aligned} p & = - \left( {a_{1} - \frac{{2a_{1} u_{1} }}{{K_{1} }} - \frac{{\alpha a_{1} u_{2} }}{{K_{1} }} + c_{1} u_{2} + a_{2} - \frac{{2a_{2} u_{2} }}{{K_{2} }} - \frac{{\beta a_{2} u_{1} }}{{K_{2} }} + c_{2} u_{1} } \right) \\ & = a_{1} - a_{2} + \frac{{\beta a_{2} K_{1} }}{{K_{2} }} - c_{2} K_{1} \\ \end{aligned}$$

$$\begin{aligned} q & = \left( {a_{1} - \frac{{2a_{1} u_{1} }}{{K_{1} }} - \frac{{\alpha a_{1} u_{2} }}{{K_{1} }} + c_{1} u_{2} } \right)\left( {a_{2} - \frac{{2a_{2} u_{2} }}{{K_{2} }} - \frac{{\beta a_{2} u_{1} }}{{K_{2} }} + c_{2} u_{1} } \right) - \left( {c_{1} u_{1} - \frac{{\alpha a_{1} u_{1} }}{{K_{1} }}} \right)\left( {c_{2} u_{2} - \frac{{\beta a_{2} u_{2} }}{{K_{2} }}} \right) \\ & = - a_{1} \left( {a_{2} - \frac{{\beta a_{2} K_{1} }}{{K_{2} }} + c_{2} K_{1} } \right) \\ \end{aligned}$$

According to Theorem (Xue 2011), since $p > 0$ and ${\text{q}} > 0$,$K_{2} < \frac{{\beta a_{2} K_{1} }}{{a_{2} + c_{2} K_{1} }}$. Therefore, the equilibrium $(u_{1}^{*} ,u_{2}^{*} ) = (K_{1} ,0)$ is stable when $K_{2} < \frac{{\beta a_{2} K_{1} }}{{a_{2} + c_{2} K_{1} }}$.

For the equilibrium

$$(u_{1}^{*} ,u_{2}^{*} ) = \left( {\frac{{a_{2} (K_{2} \alpha a_{1} - K_{2} c_{1} K_{1} - a_{1} K_{1} )}}{{ - a_{1} a_{2} + \alpha \beta a_{1} a_{2} - \beta a_{2} c_{1} K_{1} - c_{2} K_{2} \alpha a_{1} + c_{2} K_{2} c_{1} K_{1} }},\frac{{a_{1} (K_{1} \beta a_{2} - K_{2} c_{2} K_{1} - a_{2} K_{2} )}}{{ - a_{1} a_{2} + \alpha \beta a_{1} a_{2} - \beta a_{2} c_{1} K_{1} - c_{2} K_{2} \alpha a_{1} + c_{2} K_{2} c_{1} K_{1} }}} \right),$$

$$\begin{aligned} p & = - \left( {a_{1} - \frac{{2a_{1} u_{1} }}{{K_{1} }} - \frac{{\alpha a_{1} u_{2} }}{{K_{1} }} + c_{1} u_{2} + a_{2} - \frac{{2a_{2} u_{2} }}{{K_{2} }} - \frac{{\beta a_{2} u_{1} }}{{K_{2} }} + c_{2} u_{1} } \right) \\ & = \frac{{ - a_{1} a_{2} (K_{2} \alpha a_{1} - K_{2} c_{1} K_{1} - a_{1} K_{1} )}}{{ - K_{1} ( - a_{1} a_{2} + \alpha \beta a_{1} a_{2} - \beta a_{2} c_{1} K_{1} - c_{2} K_{2} \alpha a_{1} + c_{2} K_{2} c_{1} K_{1} )}} \\ & \quad + \frac{{ - a_{2} a_{1} (K_{1} \beta a_{2} - K_{2} c_{2} K_{1} - a_{2} K_{2} )}}{{ - K_{2} ( - a_{1} a_{2} + \alpha \beta a_{1} a_{2} - \beta a_{2} c_{1} K_{1} - c_{2} K_{2} \alpha a_{1} + c_{2} K_{2} c_{1} K_{1} )}} \\ \end{aligned}$$

$$\begin{aligned} q & = \left( {a_{1} - \frac{{2a_{1} u_{1} }}{{K_{1} }} - \frac{{\alpha a_{1} u_{2} }}{{K_{1} }} + c_{1} u_{2} } \right)\left( {a_{2} - \frac{{2a_{2} u_{2} }}{{K_{2} }} - \frac{{\beta a_{2} u_{1} }}{{K_{2} }} + c_{2} u_{1} } \right) - \left( {c_{1} u_{1} - \frac{{\alpha a_{1} u_{1} }}{{K_{1} }}} \right)\left( {c_{2} u_{2} - \frac{{\beta a_{2} u_{2} }}{{K_{2} }}} \right) \\ & = \frac{{a_{1} a_{2} (a_{1} K_{2} \alpha - c_{1} K_{1} K_{2} - a_{1} K_{1} )(a_{2} K_{1} \beta - c_{2} K_{1} K_{2} - a_{2} K_{2} )}}{{ - ( - a_{1} a_{2} + \alpha \beta a_{1} a_{2} - \beta a_{2} c_{1} K_{1} - c_{2} K_{2} \alpha a_{1} + c_{2} K_{2} c_{1} k_{1} )K_{1} K_{2} }} \\ \end{aligned}$$

Assuming the denominator of the equilibrium is smaller than zero, $\hbox{max} \left( {\frac{{a_{1} (c_{2} K_{2} \alpha + a_{2} - \alpha \beta a_{2} )}}{{c_{1} (c_{2} K_{2} - \beta a_{2} )}},\frac{{K_{2} \alpha a_{1} }}{{K_{2} c_{1} + a_{1} }}} \right) < K_{1} < \frac{{a_{2} K_{2} }}{{\beta a_{2} - K_{2} c_{2} }}$ or $\frac{{K_{2} \alpha a_{1} }}{{K_{2} c_{1} + a_{1} }} < K_{1} < \frac{{a_{1} (c_{2} K_{2} \alpha + a_{2} - \alpha \beta a_{2} )}}{{c_{1} (c_{2} K_{2} - \beta a_{2} )}}$ must be satisfied. Therefore, $- a_{1} a_{2} + \alpha \beta a_{1} a_{2} - \beta a_{2} c_{1} K_{1} - c_{2} K_{2} \alpha a_{1} + c_{2} K_{2} c_{1} K_{1} < 0$, $a_{1} K_{2} \alpha - c_{1} K_{1} K_{2} - a_{1} K_{1} < 0$ and $a_{2} K_{1} \beta - c_{2} K_{1} K_{2} - a_{2} K_{2} < 0$. Now, $p > 0,q > 0$, which suggests that $(u_{1}^{*} ,u_{2}^{*} ) = \left( {\frac{{a_{2} (K_{2} \alpha a_{1} - K_{2} c_{1} K_{1} - a_{1} K_{1} )}}{{ - a_{1} a_{2} + \alpha \beta a_{1} a_{2} - \beta a_{2} c_{1} K_{1} - c_{2} K_{2} \alpha a_{1} + c_{2} K_{2} c_{1} K_{1} }},\frac{{a_{1} (K_{1} \beta a_{2} - K_{2} c_{2} K_{1} - a_{2} K_{2} )}}{{ - a_{1} a_{2} + \alpha \beta a_{1} a_{2} - \beta a_{2} c_{1} K_{1} - c_{2} K_{2} \alpha a_{1} + c_{2} K_{2} c_{1} K_{1} }}} \right)$ is stable.

Assuming that the denominator of the equilibrium is greater than zero, $\frac{{a_{2} K_{2} }}{{\beta a_{2} - K_{2} c_{2} }} < K_{1} < \hbox{min} \;\left( {\frac{{K_{2} \alpha a_{1} }}{{K_{2} c_{1} + a_{1} }},\frac{{a_{1} (c_{2} K_{2} \alpha + a_{2} - \alpha \beta a_{2} )}}{{c_{1} (c_{2} K_{2} - \beta a_{2} )}}} \right)$ must be satisfied. Therefore, $- a_{1} a_{2} + \alpha \beta a_{1} a_{2} - \beta a_{2} c_{1} K_{1} - c_{2} K_{2} \alpha a_{1} + c_{2} K_{2} c_{1} K_{1} > 0$, $a_{1} K_{2} \alpha - c_{1} K_{1} K_{2} - a_{1} K_{1} > 0$, and $a_{2} K_{1} \beta - c_{2} K_{1} K_{2} - a_{2} K_{2} > 0$. Now,$p > 0,q < 0$, which suggests that equilibrium $(u_{1}^{*} ,u_{2}^{*} ) = \left( {\frac{{a_{2} (K_{2} \alpha a_{1} - K_{2} c_{1} K_{1} - a_{1} K_{1} )}}{{ - a_{1} a_{2} + \alpha \beta a_{1} a_{2} - \beta a_{2} c_{1} K_{1} - c_{2} K_{2} \alpha a_{1} + c_{2} K_{2} c_{1} K_{1} }},\frac{{a_{1} (K_{1} \beta a_{2} - K_{2} c_{2} K_{1} - a_{2} K_{2} )}}{{ - a_{1} a_{2} + \alpha \beta a_{1} a_{2} - \beta a_{2} c_{1} K_{1} - c_{2} K_{2} \alpha a_{1} + c_{2} K_{2} c_{1} K_{1} }}} \right)$ is not stable.

Based on the analysis of “Stability of equilibria in associated computational models” section and “Ecology–society computational model” section, there are two advantages for the new parameters *c*_1_ and *c*_2_, which are: (1) *c*_1_ and *c*_2_ represent the attractive force of their own languages to the competitor. Therefore, *c*_1_ and *c*_2_ show the bidirectional attractive forces of two languages, while the original model in the previous section only represents unidirectional attractive force; and (2) Equations in the new model have positive and stable equilibriums under some conditions, which provides the possibility of co-existence or even co-development for two competitive languages.

## Experiments and analysis

### Fundamental rules of determining parameter values

Based on the analysis of the stabilities of the equilibria given in the “Ecology–society computational model” section, it can be seen that the parameter values must meet stability requirements for the equilibria. The requirements are as follows:

$${\text{If}}\;c_{2} K_{2} - \beta a_{2} > 0,\;\frac{{K_{2} \alpha a_{1} }}{{K_{2} c_{1} + a_{1} }} < K_{1} < \frac{{a_{1} (c_{2} K_{2} \alpha + a_{2} - \alpha \beta a_{2} )}}{{c_{1} (c_{2} K_{2} - \beta a_{2} )}}.$$

$${\text{If}}\;c_{2} K_{2} - \beta a_{2} < 0,\;\hbox{max} \left( {\frac{{K_{2} \alpha a_{1} }}{{K_{2} c_{1} + a_{1} }},\frac{{a_{1} (c_{2} K_{2} \alpha + a_{2} - \alpha \beta a_{2} )}}{{c_{1} (c_{2} K_{2} - \beta a_{2} )}}} \right) < K_{1} < \frac{{a_{2} K_{2} }}{{\beta a_{2} - K_{2} c_{2} }}.$$

Suppose $K_{1} = 1,\;K_{2} = 1$. Taking the first case as an example, the requirement is $\frac{{a_{1} (c_{2} K_{2} \alpha + a_{2} - \alpha \beta a_{2} )}}{{c_{1} (c_{2} K_{2} - \beta a_{2} )}} > 1$, then $\alpha > \frac{{c_{1} }}{{a_{1} }}$.

### Simulation results

#### Experiments on the coexistence of two languages greatly different in strength

The following value set was chosen based on the basic rules for determining parameter values:

$$d_{1} = 0. 0 0 1 ;\;d_{2} = 0. 0 1 ;\;a_{1} = 0. 0 8 ;\;a_{2} = 0. 0 1 ; { }K_{1} = 1 ;\;K_{2} = 1 ;\;c_{1} = 0. 0 4 ;\;c_{2} = 0. 0 2 ;\;\alpha = 0. 6 ;\;\beta = 0. 6 5$$

**Fig. 1:**
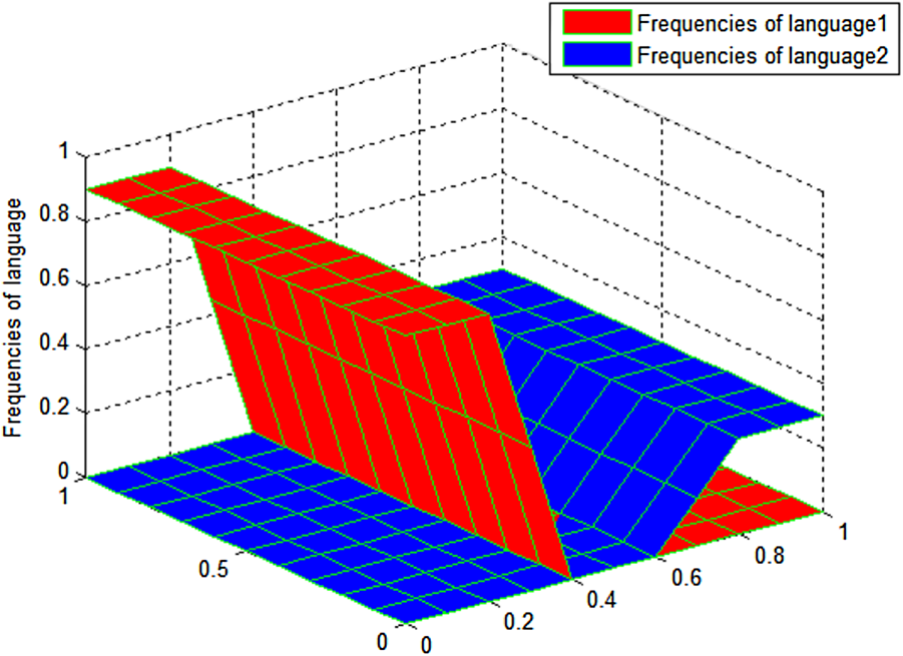
Initial value distribution of $u_{1} ,u_{2}$ for experiment on the coexistence of two languages greatly different in strength, where $u_{1}$ is the frequency of language 1 and $u_{2}$ is the frequency of language 2

**Fig. 2:**
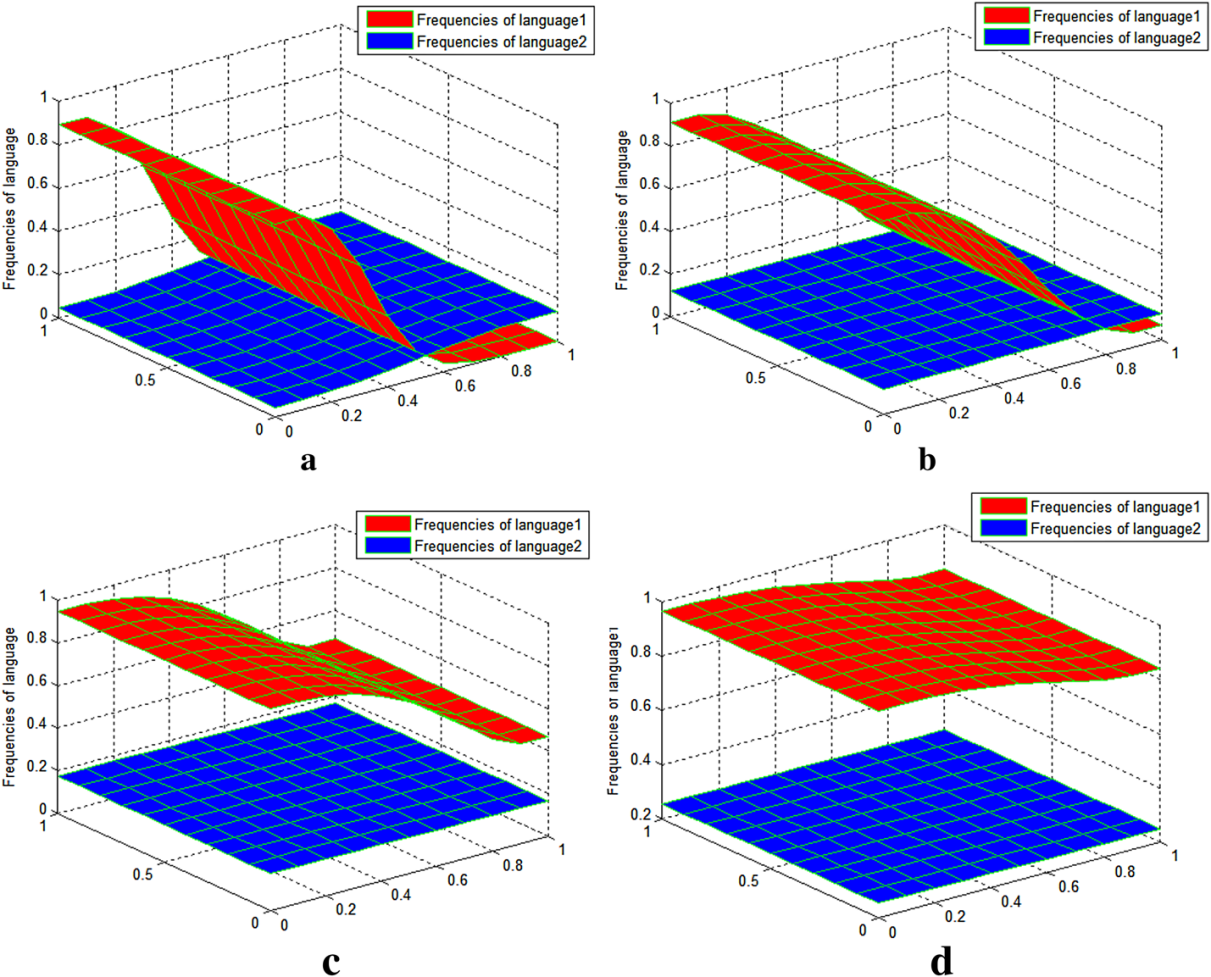
Value distribution of $u_{1} ,u_{2}$ for experiment on the coexistence of two languages greatly different in strength. Sub-image **a** t = 100, sub-image **b** t = 300, sub-image **c** t = 500 and sub-image **d** t = 700. Sub-images **a–d** show the changes in the frequency of the two languages at different moments. With the advance of observed time moment, the originally more powerful language 1 becomes even more superior. The originally weaker language 2 co-exists with language 1 without dying out

This parameter set demonstrates that language 1 has a greater advantage than language 2. Boundary conditions are $\frac{{\partial u_{1} }}{\partial n} = 0,\frac{{\partial u_{2} }}{\partial n} = 0$, where $u_{1} ,u_{2} \in \partial D$; D is a [0,1] × [0,1] rectangular area. Figure 1 shows the initial distribution of $u_{1} ,u_{2}$ for the cases of two coexistence languages greatly different in strength, which was taken from (Kandler and Steele 2008). It can be seen that the frequency of language 1 is superior to that of language 2 in its initial distribution, which is consistent with the purpose of choosing other experimental parameter values. Figure 2 shows the changes in the frequency of the two languages at different moments.

Table 1 shows the maximum and minimum values of the language frequency at different moments.

**Table 1:** Maximum and minimum value distribution of $u_{1} ,u_{2}$ at different moments for experiment 1

Values of t (min, max)	t = 0	*t* = 100	*t* = 300	*t* = 500	*t* = 700
$u_{1}$	(0.0, 0.9)	(0.0003, 0.8914)	(0.0689, 0.9093)	(0.4656, 0.9480)	(0.8399, 0.9652)
$u_{2}$	(0.0, 0.3)	(0.0466, 0.1389)	(0.1210, 0.1211)	(0.1653, 0.1757)	(0.2482, 0.2548)

It can be seen that the maximum values of $u_{1} ,u_{2}$ first decreased and then increased later, while the minimum values of $u_{1} ,u_{2}$ always increased. During observation, the weaker language, language 2, never appeared to be close to extinction and even coexisted with the stronger language, language 1.

#### Experiments on the co-development of two well-matched languages

The following values were chosen based on the basic rules for determining parameter values:

$$d_{1} = 0. 0 0 5 ;\;d_{2} = 0. 0 0 5 ;\;a_{1} = 0. 0 2 ;\;a_{2} = 0. 0 1 ;\;K_{1} = 1 ;\;K_{2} = 1 ;\;c_{1} = 0. 0 1 6 ;\;c_{2} = 0. 0 2 ;\;\alpha = 0. 6 ;\;\beta = 0. 5.$$

**Fig. 3:**
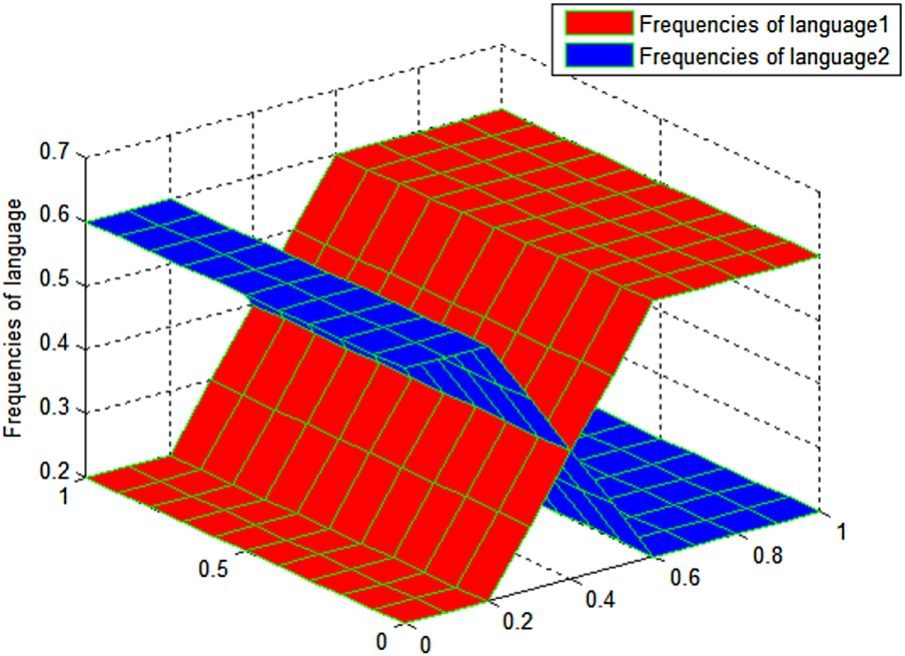
Initial value distribution of $u_{1} ,u_{2}$ for experiment on the co-development of two well-matched languages. The initial distributions of the frequency of language 1 *u*_1_ and frequency of language 2 *u*_2_ are roughly equivalent

**Fig. 4:**
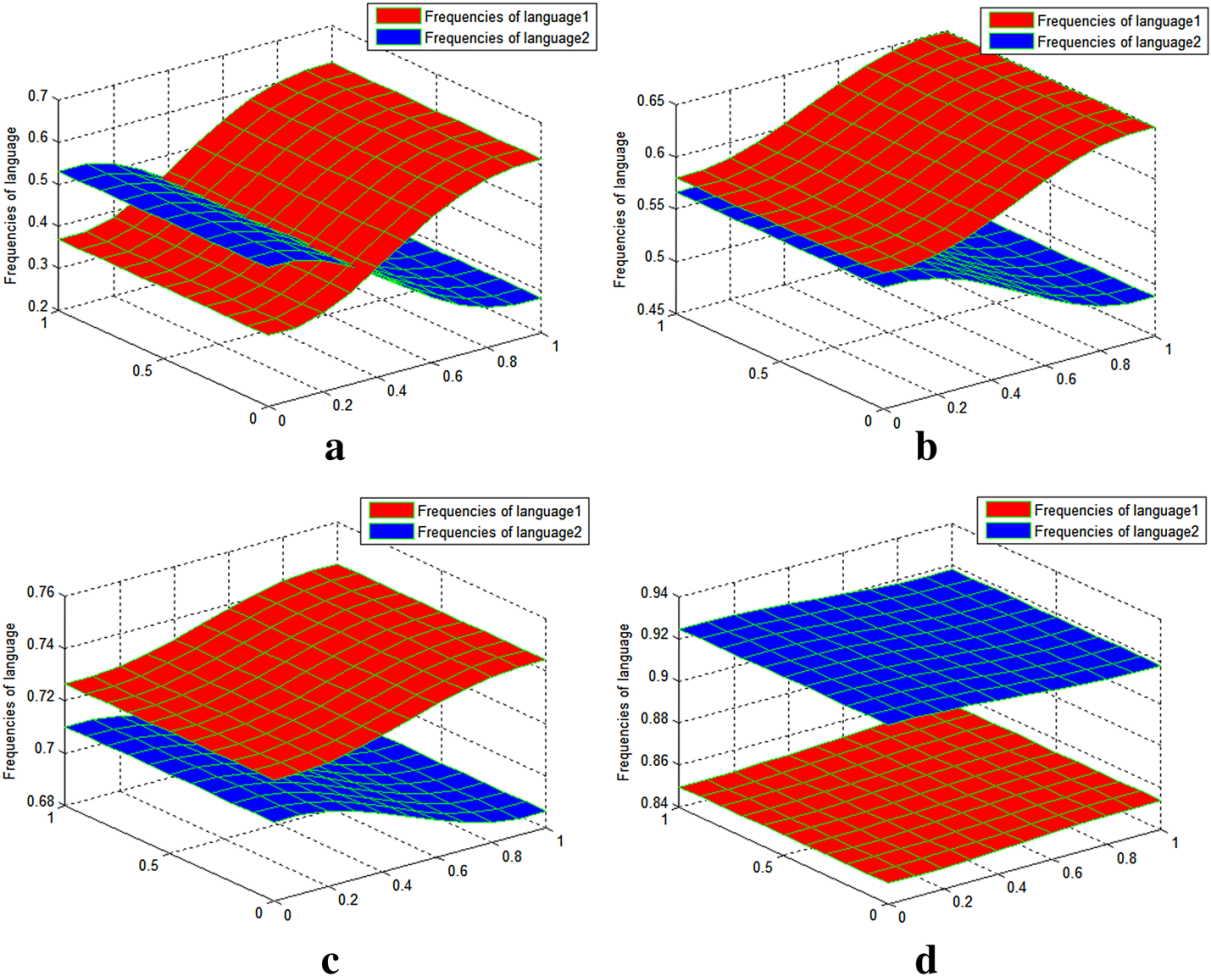
Value distribution of $u_{1} ,u_{2}$ for experiment on the co-development of two well-matched languages. Sub-image **a** t = 100, sub-image **b** t = 300, sub-image **c** t = 500 and sub-image **d**: t = 700. Sub-images **a**–**d** show the changes in the frequency of the two well-matched languages at different moments. With the advance of observed time moment, the two well-matched languages show co-development in the process of competition

These parameter values make the two languages well-matched. The boundary conditions are $\frac{{\partial u_{1} }}{\partial n} = 0,\frac{{\partial u_{2} }}{\partial n} = 0$, where $u_{1} ,u_{2} \in \partial D$; D is a [0,1] × [0,1] rectangle area. Figure 3 shows the initial distribution of $u_{1} ,u_{2}$ during the co-development of two languages that are similar in strength. It can be seen that the frequencies of languages 1 and 2 are roughly the same in their initial distributions, which is consistent with the purpose of determining the values of other experimental parameters. Figure 4 shows the changes in the frequency of the two languages at different moments.

Table 2 shows the maximum and minimum values of the frequencies of the two languages at different moments.

**Table 2:** The maximum and minimum diffusion of $u_{1} ,u_{2}$ at different moments for experiment 2

Values of t (max, min)	*t* = 0	*t* = 100	*t* = 300	*t* = 500	*t* = 700
$u_{1}$	(0.2, 0.6)	(0.3680, 0.6128)	(0.5796, 0.6494)	(0.7259, 0.7438)	(0.8491, 0.8532)
$u_{2}$	(0.2, 0.6)	(0.2817, 0.5310)	(0.4869, 0.5660)	(0.6853, 0.7093)	(0.9167, 0.9236)

It can be seen that the maximum value of $u_{1}$ increased, and the maximum value of $u_{2}$ decreased at first and then increased later. Moreover, the minimum values of $u_{1} ,u_{2}$ both increased at growth rates larger than their respective maximum values. Each language developed harmoniously within its scope, and the two languages co-developed. Languages 1 and 2 were evenly matched in general. Interestingly, language 1 was dominant in Fig. 2b, c, while language 2 was dominant in Fig. 2d Reviewing the parameter values of *c*_1_ and *c*_2_, *c*_2_ > *c*_1_, it is reasonable to conclude that language 2 is superior to language 1 in terms of social property. The analysis suggests that the social properties of language may play a more important role in language competition than previously thought.

## Conclusion

Based on an overall mathematical analysis of related language competition models, this paper has proposed an ecology–society computational model describing language competition. Key features of this ecology–society computational model are as follows. First, the model changes the assumption from previous research that one language is always superior to the other language. That is, there is only one unidirectional attractive force c of the stronger language to the weaker language. However, in the present model, the attractive forces of both *c*_1_ and *c*_2_ are introduced to cancel the indication that one language must be superior to another language. Second, this model has positive and stable equilibriums in some conditions, which provides the possibility of co-existence or even co-development for two competitive languages. Based on simulation experiments regarding the coexistence of two languages greatly different in strength and the co-development of two well-matched languages, it appears possible that two languages in competition may coexist and even develop jointly.

## References

[CR1] Abrams DM, Strogatz SH (2003). Linguistics: modelling the dynamics of language death. Nature.

[CR2] Allentoft ME, Sikora M, Sjögren KG, Rasmussen S, Rasmussen M, Stenderup J, Malaspinas AS (2015). Population genomics of Bronze Age Eurasia. Nature.

[CR3] Caridi I, Nemiña F, Pinasco JP, Schiaffino P (2013). Schelling-voter model: an application to language competition. Chaos Solitons Fractals.

[CR4] De BB, Zuidema W (2010). Multi-agent simulations of the evolution of combinatorial phonology. Adapt Behav.

[CR5] Ghirlanda S, Enquist M, Perc M (2010). Sustainability of culture-driven population dynamics. Theor Popul Biol.

[CR6] Gong T (2011). Simulating the coevolution of compositionality and word order regularity. Interact Stud.

[CR7] Haak W, Lazaridis I, Patterson N, Rohland N, Mallick S, Llamas B, Fu Q (2015). Massive migration from the steppe was a source for Indo-European languages in Europe. Nature.

[CR8] Kandler A, Steele J (2008). Ecological models of language competition. Biol Theory.

[CR9] Ke J, Ogura M, Wang WSY (2003). Optimization models of sound systems using genetic algorithms. Comput Linguist.

[CR10] Kirby S, Griffiths T, Smith K (2014). Iterated learning and the evolution of language. Curr Opin Neurobiol.

[CR11] Labov W (1994). Principles of linguistic change: internal factors.

[CR12] Labov W (2001). Principles of linguistic change: social factors.

[CR13] Mufwene SS (2001). The ecology of language evolution.

[CR14] Mukherjee A, Choudhury M, Basu A, Ganguly N (2007). Modeling the co-occurrence principles of the consonant inventories: a complex network approach. Int J Mod Phys C.

[CR15] Novembre J (2015). Human evolution: ancient DNA steps into the language debate. Nature.

[CR16] Patriarca M, Leppänen T (2004). Modeling language competition. Phys A.

[CR17] Perc M (2012). Evolution of the most common English words and phrases over the centuries. J R Soc Interface.

[CR18] Perc M (2014). The Matthew effect in empirical data. J R Soc Interface.

[CR19] Perc M, Szolnoki A (2010). Coevolutionary games—a mini review. BioSystems.

[CR20] Petersen AM, Tenenbaum JN, Havlin S, Stanley HE, Perc M (2012). Languages cool as they expand: allometric scaling and the decreasing need for new words. Sci Rep.

[CR21] Pinasco JP, Romanelli L (2006). Coexistence of languages is possible. Phys A.

[CR22] Radicchi F, Castellano C, Cecconi F, Loreto V, Parisi D (2004). Defining and identifying communities in networks. Prpc Natl Acad Sci.

[CR23] Redford MA, Chen CC, Mkkulainen R (2001). Constrained emergence of universals and variation in syllable systems. Lang Speech.

[CR24] Rilling JK (2014). Comparative primate neurobiology and the evolution of brain language systems. Curr Opin Neurobiol.

[CR25] Rilling JK, Glasser MF, Preuss TM, Ma X, Zhao T, Hu X, Behrens TE (2008). The evolution of the arcuate fasciculus revealed with comparative DTI. Nat Neurosci.

[CR26] Shaobai Z, Yanchun J, Liwen H (2015). Research on the mechanism for phonating stressed English syllables based on DIVA model. Neurocomputing.

[CR27] Stauffer D, Castelló X, Eguiluz VM, San MM (2007). Microscopic Abrams–Strogatz model of language competition. Phys A.

[CR28] Xue Y (2011). Mathematical modeling.

[CR29] Zhang M, Gong T (2013). Principles of parametric estimation in modeling language competition. Proc Natl Acad Sci.

